# Assessment of Prescriptions in the Endocrinology Department of a Tertiary Care Hospital in Pakistan Using World Health Organization Guidelines

**DOI:** 10.1155/2020/3705704

**Published:** 2020-05-30

**Authors:** Majid Khan, Rahmat Ullah, Asaf Khan, Najm ur-Rahman, Sadaqat Khan, Muhammad Riaz

**Affiliations:** ^1^Department of Pharmacy, Shaheed Benazir Bhutto University Sheringal, Dir Upper, Sheringal, Khyber Pakhtunkhwa, Pakistan; ^2^Endocrinology Ward, Lady Reading Hospital, Peshawar, Khyber Pakhtunkhwa, Pakistan

## Abstract

**Background:**

It is essential to follow World Health Organization drug prescribing indicators to ensure rational prescribing in every health care setting.

**Objective:**

To evaluate the prescriptions in the endocrinology department, according to the World Health Organization (WHO), recommended Ghana guidelines for diabetes management and rational therapy.

**Methods:**

Concurrent and retrospective study design was used. The prescriptions of 100 diabetes patients were assessed for the type of medicine, dosage form, number of drugs, diabetes type, and deviation from standard guidelines.

**Results:**

In a total of 100 prescriptions, the pattern was reported as injections (31%), antibiotics (18%), and metformin (31.1%). Half of the prescriptions were according to WHO guidelines. The number of drugs per prescription was reported at 5.2. A 70% rational approach was followed in prescribing. 81% of drugs were prescribed from the Essential Drug List (EDL) of the WHO. However, the National Essential Drug List (NEDL) was followed by 27%. The percentage of drugs on generic names was 0.7%. Eighty-four patients showed net improvement in health; 16 patients showed higher glycemic range at the time of discharge.

**Conclusion:**

The conclusion of the present study indicates that WHO Ghana guidelines were not followed up to the mark to improve the overall health status of diabetic patients and rational prescribing.

## 1. Introduction

According to the World Health Organization (WHO), diabetes mellitus is a clinical syndrome characterized by prominent symptoms of elevated blood glucose, frequent eating, excessive thirst, and frequent urination [1–3]. However, many adults have been reported as asymptomatic in diabetes; the diagnostic procedures involve monitoring of blood glucose levels both before and after the meal, which is further supported by other tests, including haemoglobin A_1C._ Recent reports indicate that 171 million people had diabetes in 2000, and this ratio promoted to 366 million in the world [1, 2].

Diabetes mellitus has three forms: Type 1 (insulin-dependent diabetes, juvenile or familial diabetes), Type 2 (maturity onset diabetes or noninsulin dependent), and gestational diabetes mellitus (GDM). The cause of diabetes mellitus includes pancreatic diseases, genetic mutations, environmental factors such as excessive intake of calories and lack of exercise, and drugs-induced diabetes, e.g., glucocorticoids, antipsychosis, and anti-AIDS drugs [1, 2].

In Pakistan, every year, half a million people die due to medication errors, which include errors in dose, medicine, and prescription writing [4]. The risk can be initially mitigated on a diet [1, 5]. The rational use of drugs can minimize medication errors if correct patient, exact medication, precise dose, exact route, factual time, and proper documentation are carried out [6, 7].

**Figure 1:**
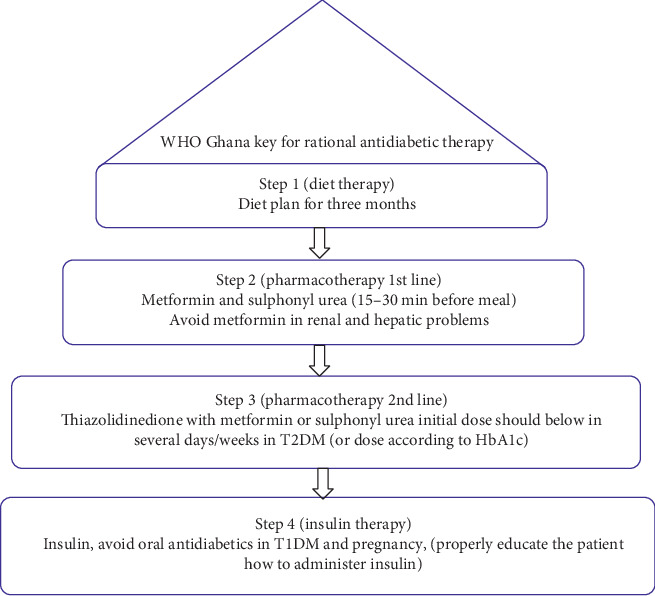
WHO Ghana key for rational antidiabetic therapy [1]. These steps are followed stepwise based on the diabetes management.

The objective of the present study is to assess prescribing indicators and to know the applicability of the WHO Ghana guidelines (Figure 1) in diabetes management. In the present study, we also assess the types of errors in prescriptions in a tertiary care hospital.

## 2. Methodology

### 2.1. Study Setting

The study was carried out in the Endocrinology Ward of the Lady Reading Hospital, Peshawar, from October 2018 to February 2019. It is an old and famous hospital of the province Khyber Pakhtun Khwa, established in 1927. The facility consists of 33 departments, 400 doctors, and 4000 support staff. The hospital has provided services in out-patient clinics to 841,199 patients and the emergency department to 1,328,336 patients and 141,991 patients admitted in 2019. The endocrinology department provides services via different subunits such as diabetes clinic, general endocrinology clinic, diabetic foot clinic, diabetes in pregnancy clinic, and insulin bank services [8].

### 2.2. Study Design

A cross-sectional observational study design was used. The prescriptions of all admitted patients to the endocrinology department were considered irrespective of age, gender or race, etc.

### 2.3. Inclusion Criteria

All admitted diabetic patients in the endocrinology department were included in the study.

### 2.4. Exclusion Criteria

All nondiabetic and admitted patients of less than one day were excluded.

### 2.5. Prescribing Indicators

Prescriptions to manage diabetes mellitus were assessed using key WHO indicators. According to the WHO Ghana guidelines as given in Figure 1, the average number of drugs per prescription, % of the generic name, % of antibiotic prescribed, % of injection in prescription, and % of drug prescribed from the Essential Drug List (EDL) and National Essential Drug List (NEDL) were evaluated.

### 2.6. Operational Definitions

There are two types of errors in prescription classified by the WHO.

### 2.7. Omission Errors

Omission errors include errors related to the patient's biodata, such as name, address, age, and gender.

### 2.8. Commission Errors

The errors are related to the prescriber biodata such as name, address, and also errors in dose, duration, dosage form, frequency, and strength.

### 2.9. Drug Interactions

The possible interaction was assessed using drugs.com and the drug interaction checker in the British National Formulary and “Stockley's Drug Interactions” Pharmaceutical Press, 7^th^ edition.

### 2.10. Data Collection

The data were collected via special Proforma generated by the Department of Pharmacy, Shaheed Benazir Bhutto University, Sheringal, KPK, Pakistan, on the pattern recommended by the World Health Organization. Students were guided on “how to investigate drug use in health facilities,” an ethical perspective of the study and how to participate in ward rounds. The students were sent officially to the Lady Reading Hospital, Peshawar. At the end of the data collection, the approval certificates were issued to the students.

### 2.11. Data Analysis

The software MS Word for writing, MS Excel for tabulating and graphical representation of the data, the Pharmapedia mobile application, pharma guide, and British National Formulary were used to know brand names and drug-drug interactions. Basic statistics such as mean and percentage were calculated using Microsoft Excel.

### 2.12. Ethical Considerations

Ethical approval was obtained from the Pharmacy Research Ethics Committee (PREC) at the Shaheed Benazir Bhutto University, Sheringal (reference: sbbu/Pharm2018/ec dated August 2018). There was no direct involvement of the patients to take their consent about the study.

## 3. Results

### 3.1. Prescribing Indicators

In the present study, 54% of prescriptions were male. The prescriptions were evaluated using the WHO Ghana guidelines. As per the Ghana WHO guidelines, diabetes shall be controlled by diet, so 2% of the patients were managed on a diet, the second step is given in Figure 1, 34% of prescriptions were according to Step 2 of guidelines, and none of the prescriptions were according to Step 3. In comparison, Step 4 was followed in 56% of prescriptions. The percentagewise age distribution of diabetic patients is given in Table 1. Diabetes was found common in the age group of 50 to 60 years (33%). The details of the prescribing indicators are given in Table 2. The common drugs per prescription were recorded to be 5.2. 18% of antibiotics and 31% injectables were prescribed in our study. The antidiabetic drugs were prescribed as metformin (31.19%), followed by metformin + glimepiride (2.75%), glimepiride (1.8%), and gliclazide (0.9%).

Prescription errors have recorded in Table 3 as omission and commission errors that include errors related to patient history and duration of treatment.

The drug-drug interaction errors (18%) of three categories (minor, moderate, and major) have been noticed in the study; the details are given in Table 4. The minor interaction between aspirin + frusemide, dexamethasone + omeprazole, and insulin + frusemide has been noticed, and the details of interaction and effects are given for moderate and major interactions in Table 4.

## 4. Discussion

Rational prescribing is the key to quality health. The World Health Organization has developed a quality policy for rational drug treatment of diseases. In the present study, the WHO guidelines were used to evaluate the prescription in tertiary care hospitals to minimize prescription errors. Rational approach of the WHO in prescriptions is not successfully implemented, and deficiencies are still there that need to be addressed, the findings were also compared with other standards, and the element of polypharmacy was found in most of the prescriptions [9]. The WHO Ghana guidelines were designed for developing countries that are usually facing the same conditions of health and other problems. We found that diet step was followed in a minimal number of prescriptions and perhaps the conditions of such patients are not worrisome to visit a tertiary care hospital. Ghana guidelines Step 2 were followed in 34% of prescriptions. None of the prescriptions was found in Step 3 combinational therapy for diabetes; however, fourth step-based results were 36%, mostly insulin. As most of the patients visited or referred after complications that cannot be treated or managed at primary care health setup, thus the fourth step is followed comparatively more than the other three steps.

As per the WHO guidelines, the number of items per prescription will be 1.6–1.8; in our findings, the result is quite higher (5.2), is however similar to that reported in Nigeria (5.2), and is more than that in Pakistan (4.5) drugs per prescription, Iran (3.07), Zimbabwe (1.3), and Malawi (1.8), but it is lower than that reported by Hussain et al. (7.05) in Pakistan [9–12]. The higher number of drugs prescribed per prescription leads to polypharmacy, which ultimately creates issues of side effect, drug-drug interaction, drug-disease interaction, financial burden, and irrational use of drugs. It is established that polypharmacy is linked to clinical consequences [13]. As diabetes is mostly related to elderly patients, the higher number of drugs that may contain unnecessary drugs leads to an increase in morbidity and mortality that need to be minimized [14]. Thus, medication not matching diagnosis should be avoided.

Prescribed injectables (31%) in our study have crossed the WHO limits given for injectables (13.4–24.1%); the deviation has also reported in Cambodia (57.6%) [15] and in Ghana (80%) [16]. In comparison, an Indian study has been reported to fall within the WHO standards (12.27%) [17]. It is well known that overuse of injectable is an irrational and leading cause of infectious diseases such as hepatitis C and AIDS [18, 19].

The number of antibiotics as per the WHO per prescription should be less than 30%; in our study, that is 18% much better and less than Nepal (43%) [20], India (44.8%) [15], and Bangladesh (25%) [21]. The excessive use of antibiotics leads to the development of resistance.

The WHO-recommend prescriptions of drugs by the generic name should be 100%. Still, we found only 0.7 % of drugs prescribed by the generic name which is much lower than reported in other countries like Dubai (4%), Kenya (40%), Brazil (70.4%), and Ethiopia (98.7%). Not following the WHO guidelines related to generic prescribing ultimately leads to irrationality [22–24]. The inclination to prescribe by the trade name may lead to the high cost and low quality, which might be overcome if prescribed by the generic name.

The deviation from an ideal prescription was found. The percentage of missing the patient name is 5.4% in a study carried out in Saudi Arabia [25], and it is 11% in India while in our study, it is 9.17%. Information about age should be present on every prescription. Still, we found that 7% of prescriptions do not have this information, which is comparatively less than that reported in prescriptions in Saudi Arabia (22.7%) and India (10%) [25, 26]. The information about gender, weight, and diagnosis has been recorded to a satisfactory level in the present study; however, the same information has not been recorded in studies reported earlier in Saudi Arabia and India [25, 27, 28]. The lack of proper information on prescription indicates how much time was given to the diagnosis, thus leading to irrational prescribing and medication errors.

The drug-drug interaction errors (18%) of three categories (minor, moderate, and major) have been noticed in the study. The drug interactions reported in the present study are categorized into three categories: minor, moderate, and major. Minor interaction includes potassium and liver enzyme level elevation, and moderate interaction includes peptic ulceration and hypoglycemia. The major interactions include severe hypoglycemia, bleeding, and steroid toxicity.

## 5. Conclusion

The study shows deviation from rational prescribing in the study setting. The resulting values of prescribing indicators show divergence from the WHO norms. However, the percentage of encounter in prescribing antibiotics was within an optimal range. The majority of prescriptions were not according to prescription writing protocols. Based on this study, a policymaker can work for improving prescribing practice.

### 5.1. Limitation of the Study

This study was conducted only in the endocrinology department on the ward level.

### 5.2. Recommendations

- We recommend the study to be conducted in every medical setup for rational drug use and patient improvement.
- The WHO Ghana guidelines should be used as a key to managing diabetes mellitus in every healthcare.
- The policy of the healthcare setup may be revised to engage pharmacists at the ward level to minimize drug-drug interactions.
- The e-prescribing system is recommended to minimize the prescription errors and maintain record history of each patient.

## Figures and Tables

**Table 1:** Agewise distribution of patients.

Age groups (year)	01–10	10–20	20–30	30–40	40–50	50–60	60–70	70–80	80–90	Distribution
Male	04	08	06	05	06	16	04	02	01	52
Female	01	14	04	04	04	16	02	02	00	47
Total	05	22	10	09	10	33	06	04	01	100

NM = not mentioned.

**Table 2:** The standard prescribing indicators (*N* = 100).

Indicator	Value (%)	Total	Optimal level (%)
The average number of drugs per encounter	5.2	520/100	1.6–1.8
% of drug prescribed generic name	0.7	04/520	100
% of encounter antibiotics	18	98/520	20.0–26.8
% of encounters with an injection	31	36/116	13.4–24.1
% of drugs the from Essential Drug List	81	89/109	100
% of drugs from the National Essential Drug List of Pakistan	27	30/109	100
% of drug-drug interaction	18.3	20/109	0
% of pharmacoeconomic insulin	15.4	94/111	100

**Table 3:** Commission and omission errors (*N* = 100).

Type of error	No. of prescriptions contains error	Percentage (%)
Drug name error	10/109	9.17
Dosage form (NM)	20/100	3.84
Strength of the dosage form (NM)	67/520	12.8
Duration of therapy (NM)	00	00
Frequency (NM)	57/520	10.9
Patient name (NM)	01	01
Patient name duplicated	02	02
Patient address (NM)	13	13
Age (NM)	07	07
Gender (NM)	00	00
Weight (NM)	01	01
Diagnosis (NM)	00	00
Total	178	60.7

NM = not mentioned.

**Table 4:** Drug-drug interactions.

Degree of interaction	Interacting combination	Frequency (%)	Consequences
Minor	Aspirin + frusemide	4 (3.6)	Combination effect level of potassium in serum affects CYP3A4 enzyme in liver antagonize hypoglycemic effect
	Dexamethasone + omeprazole	2 (1.8)	
	Insulin + frusemide	6 (5.5)	
Moderate	Glimepiride + aspirin	1 (0.9)	Hypoglycemia chances
	Dexamethasone + aspirin	1 (0.9)	Peptic ulceration chances
	Dexamethasone + clopidogrel	1 (0.9)	Affects CYP3A4 enzyme in the liver
	Dexamethasone + insulin	1 (0.9)	Antagonize hypoglycemic effect
Major	Metformin + metformin	1 (0.9)	Severe hypoglycemia chances
	Heparin + aspirin + toradol	1 (0.9)	Bleeding chances
	Dexamethasone + hydrocortisone	1 (0.9)	Steroid toxicity chances (cushing syndrome)
	Dexamethasone + triamcinolone	1 (0.9)	
Total		20 (18.1)	

## Data Availability

The data used to support the findings of this study are available from the first and corresponding authors upon request.
